# The proposed biosynthesis of procyanidins by the comparative chemical analysis of five *Camellia* species using LC-MS

**DOI:** 10.1038/srep46131

**Published:** 2017-04-06

**Authors:** Liang Zhang, Yuling Tai, Yijun Wang, Qilu Meng, Yunqiu Yang, Shihua Zhang, Hua Yang, Zhengzhu Zhang, Daxiang Li, Xiaochun Wan

**Affiliations:** 1 State Key Laboratory of Tea Plant Biology and Utilization, Anhui Agricultural University, Hefei 230036, China; 2 School of Life Science, Anhui Agricultural University, Hefei 230036, China

## Abstract

The genus *Camellia (C*.) contains many species, including *C. sinensis, C. assamica*, and *C. taliensis, C. gymnogyna* and *C. tachangensis*. The polyphenols of *C. sinensis* and *C. assamica* are flavan-3-ols monomers and their dimers and trimmers. However, the biosynthesis of procyanidins in *Camellia* genus remains unclear. In the present study, a comparative chemical analysis of flavan-3-ols, flavan-3-ols glycoside and procyanidins was conducted by high performance liquid chromatography (HPLC) and liquid chromatography diode array detection coupled with triple-quadrupole mass-spectrometry (LC-DAD-QQQ-MS). The results showed that *C. tachangensis* had a significant higher contents of (-)-epicatechin (EC) and (-)-epigallocatechin (EGC) compared with *C. sinensis* (p < 0.001). By contrast, higher levels of galloylated catechins were detected in *C. sinensis*. LC-DAD-MS/MS indicated that the main secondary metabolites of *C. tachangensis* were non-galloylated catechins, procyanidin dimers and trimers. Furthermore, (-)-epicatechin glucose (EC-glucose) and (-)-epigallocatechin glucose (EGC-glucose) were also abundant in *C. tachangensis*. A correlation analysis of EC-glucose and procyanidins dimers was conducted in five *Camellia* species. The levels of EC-glucose were closely related to the procyanidin dimers content. Thus, it was suggested that EC-glucose might be an important substrate for the biosynthesis of procyanidins.

The genus *Camellia* (C.) is a large family of plants that originated in South-western China and includes hundreds of species. Two famous *Camellia* species, *Camellia sinensis* (L.) O. Ktze and *Camellia assamica* (Mast.) Chang, are cultivated worldwide for tea production. In addition, *C. tachangensis* is also used as the raw material for tea in Yunnan province, China^1^. The main secondary metabolites of *C. sinensis* are flavan-3-ols and their gallate esters^2^. These compounds are mainly distributed in the leaves of *C. sinensi*s^3^,^4^. (-)-Epigallocatechin gallate (EGCG) is a typical flavan-3-ol in the leaves of *C. sinensis*, whose biosynthetic pathway has been clarified^5^. It was reported that galloyl-1-O-*β*-D-glucosyltransferase and 1-O-galloyl-*β*-D-glucose O-galloyltransferase were the critical enzymes in the biosynthesis of galloylated catechins^5^. Furthermore, procyanidins are distributed abundantly in the roots of *C. sinensis*^6^. However, the biosynthesis of procyanidins in *Camellia* species has not been clarified.

In 1980s, *C. tachangensis* was first identified in Yunnan, China. It was thought that *C. tachangensis, C. taliensis and C. gymnogyna* are different from *C. sinensis* by chloroplast genome sequences analysis. During the domestication process of *Camellia* species, *C. sinensis* has become the optimal species to produce tea products, such as green tea. To study the relationships of various *Camellia* species, taxonomic approaches have been applied^7^. The taxonomy of *Camellia* species relied on the traditional morphology-based classification. However, recently, some molecular markers have been tentatively used in taxonomic studies^8^. Moreover, chemotaxonomy is also a potent tool to study the relationships among *Camellia* species^9^. To understand the value of *Camellia* species, more attention should be paid to the taste and flavour chemistry of their leaves. The taste and bioactivities of green tea were attributed to a complicated combination of L-theanine, catechins, and caffeine^10^,^11^. For example, condensed tannins have an intensely astringent taste. Therefore, they are usually artificially decreased in tea plantations^12^,^13^. The chemical units of the procyanidins are usually EC and EGC^13^. Thus, it was deduced that the biosynthesis of procyanidins derives from the auto-polymerisation of flavan-3-ols monomers^14^. However, the biosynthesis of procyanidins in *Camellia* plants remains unclear.

Liquid chromatography coupled mass spectroscopy (LC-MS) is a high throughput and unbiased tool that has been used to identify trace secondary metabolites of plants^15^. To study the chemical characteristics among various *Camellia* species, metabolomics analysis combined with LC-MS has been applied to identify the differences between wild-types and cultivars of *Camellia* species (unpublished data). These metabolomics analyses suggested that procyanidins might be the significant different metabolites of different *Camellia* species. To study the chemical differences of various *Camellia* species, we collected the mature leaves of C. *sinensis*, C. *assamica, C. taliensis, C. gymnogyna* and C. *tachangensis* in the same place at the same time, and performed a comprehensive quantitative analysis of the major polyphenols, procyanidins and their probable substrates.

In the present study, the contents of catechins in the mature leaves of C. *sinensis*, C. *assamica, C. taliensis, C. gymnogyna* and C. *tachangensis* were determined by HPLC. The unique secondary metabolites of C. *tachangensis* were identified by LC-DAD-MS^16^. The critical substrates involved in the biosynthesis of galloylated catechins and procyanidins were compared^17^. A proposed biosynthetic pathway for procyanidins was deduced using a correlation analysis.

## Results

### Differences in the compounds of *C. assamica, C. sinensis, C. taliensis, C. gymnogyna* and *C. tachangensis* by LC-DAD-MS/MS

**Figure 1:**
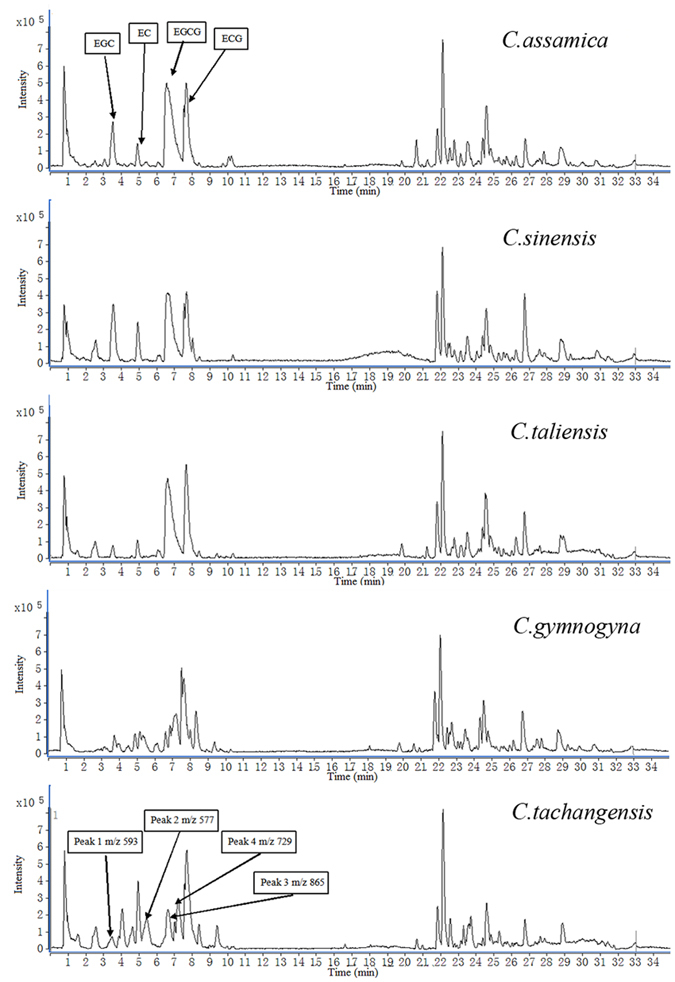
The TICs of *C. assamica, C. sinensis, C. taliensis, C. gymnogyna* and *C. tachangensis*. EGC, (-)-epigallocatechin; EC, (-)-epicatechin; EGCG, (-)-epigallocatechin gallate; ECG, (-)-epicatechin gallate.

Figure 1 shows that the total ion chromatography (TIC) profiles of C. *sinensis, C. assamica and C. taliensis* were similar, but the profiles of *C. gymnogyna* and *C. tachangensis* showed significant difference compared with other three *Camellia* samples. In the TIC of *C. tachangensis*, four unique compounds with *m/z* at 577, 593, 729 and 865 were detected. In the TICs of *C. assamica, C. sinensis* and *C. taliensis*, the catechins including EC, EGC, (-)-epicatechin gallate (ECG) and EGCG were also identified.

To explore the chemical structures of these four unique compounds in *C. tachangensis*, the ultraviolet (UV) spectrum and MS/MS spectrum of individual compound was analyzed. Compounds 1–4 showed strong absorbance intensity at the detection wavelength of 278 nm. The UV spectrum of these compounds was similar, having maximum absorption wavelengths at 230 and 278 nm (Supplementary Figure 1). These compounds might contain the flavan-3-ol unit, the maximum absorption wavelengths of which are also about 230 and 278 nm.

**Figure 2:**
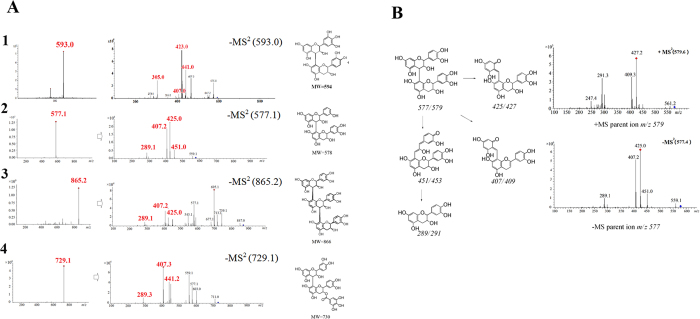
The MS/MS fragment ions of peak 1–4. (**A**), the parent ion and fragment ions of compound 1–4; (**B**), the fragmentation pathway of compound 2.

Compound 2 (peak 2) had a molecular weight of 578.1, which was deduced by the quasi-molecular ion peak of [M − H]^−^ at *m/z* 577.1. The molecular formula was calculated as C_30_H_26_O_12_. Under the MS/MS mode, peak 2 showed that [M − H]^−^ at *m/z* 577.1 produced five major secondary fragments (MS^2^) at *m/z* 559.1, 451.0, 425.0, 407.2, and 289.1 (Fig. 2). Compound 2 was identified as a procyanidins dimer (EC-EC). Furthermore, compounds 1, 3 and 4 gave [M + H]^+^ and [M − H]^−^ parent ions at *m/z* at 595/593, 867/865 and 731/729, respectively. The molecular weights of compounds 1, 3 and 4 were deduced to be 594, 866 and 730. With reference to the published mass spectra of procyanidins in tea plants, compounds 1, 3 and 4 were identified as procyanidin trimers and dimers, constructing by EGC-EC, EC-EC-EC and ECG-EC respectively^18^,^19^.

Loss of cleavage analysis provided structural information. The loss of a 126 Da fragment was the cleavage of the A-ring of flavan-3-ols. The loss of a 152 Da fragment was from the retro-Diels-Alder (RDA) fragmentation of the B-ring of flavan-3-ols. The loss of a 170 Da fragment might be derived from the continuous loss of OH after the RDA cleavage. Therefore, the neutral loss of 126, 152, and 170 could be used as the main fragmentation markers of procyanidins to analyse their chemical structures.

### The contents of catechins, gallic acid and caffeine in *C. assamica, C. sinensis, C. taliensis, C. gymnogyna* and *C. tachangensis* by HPLC

Catechins and galloylated catechins are the main secondary metabolites of *C. sinensis* and *C. assamica*^2^. According to the published method^16^, the levels of the main compounds in the five *Camellia* samples were determined. Table 1 showed that the levels of galloylated catechins, including and ECG and EGCG in *C. sinensis* and *C. assamica* were significantly higher than those in *C. tachangensis* (p < 0.001). Furthermore, the contents of caffeine in *C. tachangensis* were also significantly lower than those of *C. sinensis* and *C. tachangensis* (p < 0.001).

An important finding was that the contents of EC and EGC in *C. tachangensis* were significantly higher than those of *C. sinensis* and *C. assamica*. Usually, EGCG is the major polyphenol in the fresh leaves of *C. sinensis* and *C. assamica*^20^. In the present study, the EC and EGC content of *C. tachangensis* was about two-fold higher than that in *C. sinensis*. Even so, the amount of galloylated (-)-epicatechin in *C. tachangensis* was not increased in proportion, which means that the biosynthesis of galloylated catechins was decreased in the leaves of *C. tachangensis*^21^,^22^. These results suggested that the main polyphenols compounds in *C. tachangensis* were different from *C. sinensis* and *C. assamica*.

### The quantitative analysis of procyanidins, EC-glucose, EGC-glucose by LC-QQQ-MS

**Figure 3:**
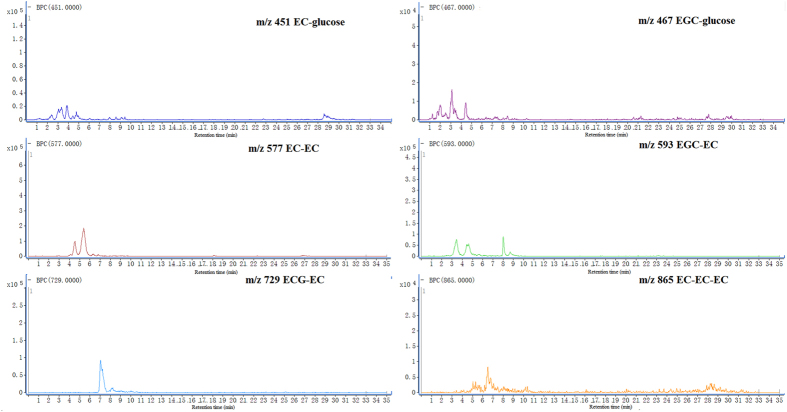
The targeted parent ions of EC-glucose, EGC-glucose, EC-EC, EGC-EC, ECG-EC and EC-EC-EC in *C. tachangensis*.

As shown in Fig. 3, the *m/z* at 577, 593, 729 and 865 were targeted in the TIC of mature leaves extract of *C. tachangensis*. Furthermore, the *m/z* at 451 and 467 were also identified as the EC-glucose and EGC-glucose by MS/MS spectrum (Supplementary Figure 2). The fragment ions of *m/z* at 451 and 467 showed main fragment ions of 289.0 ([M − H]^−^ of catechin) and 305.0 ([M − H]^−^ of gallocatechin) by losing the neutral loss 162 Da [glucose-H_2_O].

**Figure 4:**
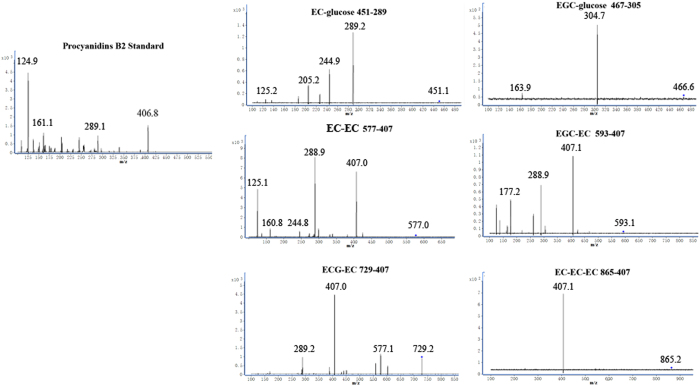
The parent ions and product ions of mass spectra (MS/MS) of EC-glucose, EGC-glucose, EC-EC, EGC-EC, ECG-EC and EC-EC-EC at the 20 V collision energy of LC-QQQ-MS.

Through the LC-QQQ-MS, the product ions of procyanidins, EC-glucose and EGC-glucose were obtained. All of the four procyanidins compounds showed a same product ion *m/z* at 407, which is a critical fragment of procyanidins. On the other side, the EC-glucose and EGC-glucose showed the main product ions *m/z* at 289 and 305, which are the [M − H]^−^ ions of EC and EGC, respectively. The collision energies (CE) were selected at 20 V for EC-glucose, EGC-glucose, procyanidins EC-EC, procyanidins EGC-EC, procyanidins ECG-EC and procyanidins EC-EC-EC. As shown in Fig. 4 multiple reaction monitoring (MRM) mode was employed to detect the target compounds by selected product ions from the parent ions (EC-glucose, 451 → 289; EGC-glucose, 467 → 305; EC-EC, 577 → 407; EGC-EC, 593 → 407; ECG-EC, 729 → 407 and EC-EC-EC, 865 → 407).

**Figure 5:**
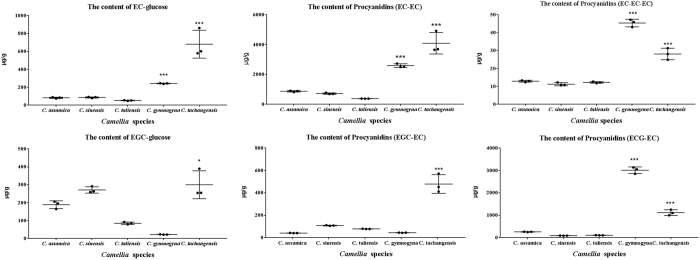
The contents of EC-glucose, EGC-glucose, EC-EC, EGC-EC, ECG-EC and EC-EC-EC in *C. assamica, C. sinensis, C. taliensis, C. gymnogyna* and *C. tachangensis*. *p < 0.05, ***p < 0.001 compared with *C. sinensis*.

Firstly, the calibration curve of procyanidins B_2_ was established (Supplementary Figure 3). The regression coefficient (r) of linear equation indicated that the LC-QQQ-MS was with good linearity for the determination of analytes. The levels of procyandins, EC-glucose and EGC-glucose were caculated. As shown in Fig. 5, the content of EC-glucose in *C. tachangensis* was significantly higher than other *Camellia* species (p < 0.001). The content of EC-glucose was 681.19 ± 156.35 μg/g in *C. tachangnesis*, compared with the 242.18 ± 3.75 μg/g, 52.03 ± 3.57 μg/g, 86.45 ± 7.50 μg/g and 82.32 ± 7.50 μg/g in *C. gymnogyna, C. taliensis, C. sinensis* and *C. assamica*. Correspondingly, the main procyanidins B_2_ in *C. tachangensis* was 4090.69 ± 719.86 μg/g, which was significantly higher than those in *C. gymnogyna* (2589.89 ± 124.03 μg/g), *C. taliensis* (380.08 ± 5.94 μg/g), *C. sinensis* (722.57 ± 47.51 μg/g) and *C. assamica* (872.29 ± 43.45 μg/g) (p < 0.001).

**Figure 6:**
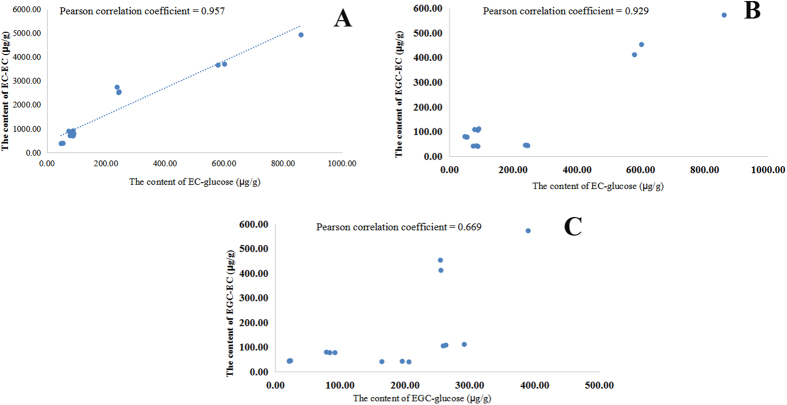
The correlation coefficient of the contents of EC-glucose and procyanidin EC-EC (**A**), EGC-glucose and procyanidin EGC-EC (**B**), EC-glucose and procyanidin EGC-EC (**C**) in five *Camellia* species.

To calculate the pearson’s correlation coefficient of the contents of EC-glucose and procyanidins B_2_, the contents of EC-glucose and EC-EC were profiled as shown in Fig. 6. With the increasing amounts of EC-glucose, the contents of procyanidins dimers (EC-EC) increased correspondingly.

Except for the procyanidins B_2_ (EC-EC), a procyanidins dimer consisting of EGC and EC unit was also identified and compared in five *Camellia* species. The EGC-glucose was also simultaneously determined with the contents of procyanidins EGC-EC. The mean value of the contents of EGC-glucose in five *Camellia* species were listed in order as *C. tachangensis* > *C. sinensis* > *C. assamica* > *C. taliensis* > *C. gymnogyna*, while the contents of EGC-EC were *C. tachangensis* > *C. sinensis* > *C. taliensis* > *C. gymnogyna* > *C. assamica*. These results suggested that the correlation of EGC-glucose and EGC-EC was weak, which was also confirmed by the pearson correlation coefficient = 0.669. Based on the procyanidins biosynthesis hypothesis, the results supposed that the contents of EC-glucose were the limiting factor for the biosynthesis of EGC-EC.

### Quantificational Real-time Polymerase Chain Reaction (qPCR) Analysis

By homology analysis, we found LAR and ANR genes in C. sinensis shared a high homology (>98%) with that in *C. tachangensis* (Supplementary Figure 4). And this result showed that the primers designed for *C. sinensis* is available for *C. tachangensis*. The amplification efficiency of these primers were added in Supplementary Figure 5. Designed primers of each gene were specific, and amplification efficiency were between 90 and 110%, so these primers were considered acceptable for qPCR reactions. Leucocyanidin reductase (LAR) (EC 1.17.1.3) and anthocyanidin reductase (ANR) (EC 1.3.1.77) are the main enzymes responsible for the biosynthesis of catechin and epicatechin, respectively^23^,^24^. Traditionally, flavan-3-ol has been considered the product of LAR, which converts leucocyanidin to catechins, after which an epimerase converts catechin to epicatechin^25^. During the biosynthesis of flavan-3-ols, anthocyanidin synthase and ANR catalyse the conversion of leucodelphinidin to (-)-epigallocatechin *via* delphinidin and/or leucocyanidin to (-)-epicatechin via cyanidin^26^. Therefore, high expressions of ANR and LAR are closely related to the high synthesis of epicatechin and catechin. In the present study, two genes in the multi-gene family of LAR (LAR1 and LAR2) and ANR (ANR1 and ANR2) were analysed by qPCR, respectively. The results showed that the relative expressions of LAR1 and LAR2 were higher in cultivars of *C. sinensis* compared with those in *C. tachangensis*. The relative expression of LAR1 in *C. sinensis* was one thousand times higher than that in *C. tachangensis*. The higher gene expression level might result in higher contents of the main flavan-3-ols (GC, gallocatechin) in *C. sinensis* compared with those in *C. tachangensis*.

**Figure 7:**
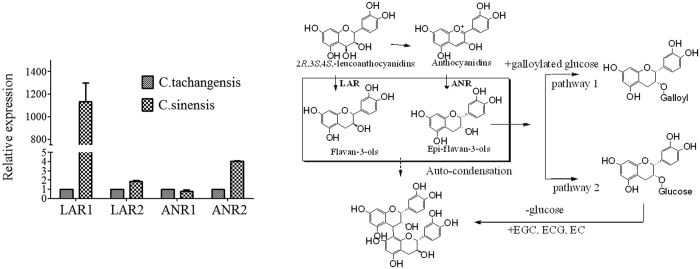
Expression of representative flavonoid biosynthetic genes, as analysed by qPCR.

By contrast, the ANR1 and ANR2 expression didn’t show significant differences between *C. sinensis* and *C. tachangensis*. This suggested that the biosynthesis of epi-flavan-3-ols was similar in these species. Similarly high contents of epi-flavan-3-ols in *C. sinensis* and *C. tachangensis* confirmed this result. However, the main epi-flavan-3-ol of *C. tachangensis* was EC and EGC, while that in *C. sinensis* was EGCG and ECG. The LC-TOF-MS analysis suggested that the down-stream metabolism of (-)-epicatechin has two pathways (Fig. 7). One pathway is the classical gallic esterification, which produces galloylated (-)-epicatechin. The other is the glycosylation of (-)-epicatechin, which would contribute to the biosynthesis of procyanidins.

## Discussion

### The chemical characteristics and secondary metabolism of *Camellia* species

Liquid chromatography time-of-flight mass spectrometry (LC-TOF-MS) and LC-DAD-MS/MS are suitable to study the chemical compounds of different *Camellia* species, whose secondary metabolites are structurally similar. Some metabolomics analyses have used LC-TOF-MS in comparative chemical study of green tea or tea leaves under different processing conditions^27^. They provided much useful information on the trace secondary metabolites. In a preliminary study, we found that the differences between *C. tachangensis* and *C. sinensis* were obvious, and we would not need to use metabolomics analysis.

The flavonoids pathway is a classical metabolic pathway in plant secondary metabolism. Traditionally, the clarification of plant secondary metabolism has relied on interpreting critical catalysing enzymes and molecular biological verification. In this process, the first step is to separate and purify the enzymes from the plant tissue, and then verify the exclusive functional reactions using related substances. This is a basic tool to study plant secondary metabolism^5^. In the present study, we attempted to elucidate the relationships of secondary metabolites using LC- MS.

In the present study, three types of secondary metabolites (phenolic acids, flavan-3-ols and flavonoids glycosides) were monitored and quantitatively analyzed using LC-QQQ-MS (Supplementary Figure 4). Firstly, the main secondary metabolites of phenylpropanoid pathway were compared in five *Camellia* species. Galloylated glucose and galloylated quinic acid were assessed^28^. These compounds are the main galloylated derivatives for the transportation and storage of gallic acid. The content of galloylated quinic acid in *C. tachangensis* were significantly higher than those in *C. assamica* and *C. sinensis*, and the level of galloylated glucose of *C. tachangensis* was also significantly higher than *C. sinensis*. In a recently published article, it was verified that galloylated glucose is the main substance for the biosynthesis of galloylated catechins^5^. Furthermore, the results suggested that galloylated quinic acid might also be involved in this process.

Secondly, the flavonoids glycosides are also major secondary metabolites of flavonoids pathways. They are distributed widely in many angiosperm plants^29^. In tea plants, over one hundred flavonoid glycosides have been identified^30^. In the present study, six typical kaempferol and quercetin glycosides were determined. As shown in Supplementary Figure 6, the contents of two falvonoids mono-glycosides (kaempferol glucose-rhamnose and quercetin glucose-rhamnose) in *C. tachangensis* were significantly higher than *C. sinensis*, while the flavonoids di-glycosides and tri-gylcosides didn’t show significant differences.

**Figure 8:**
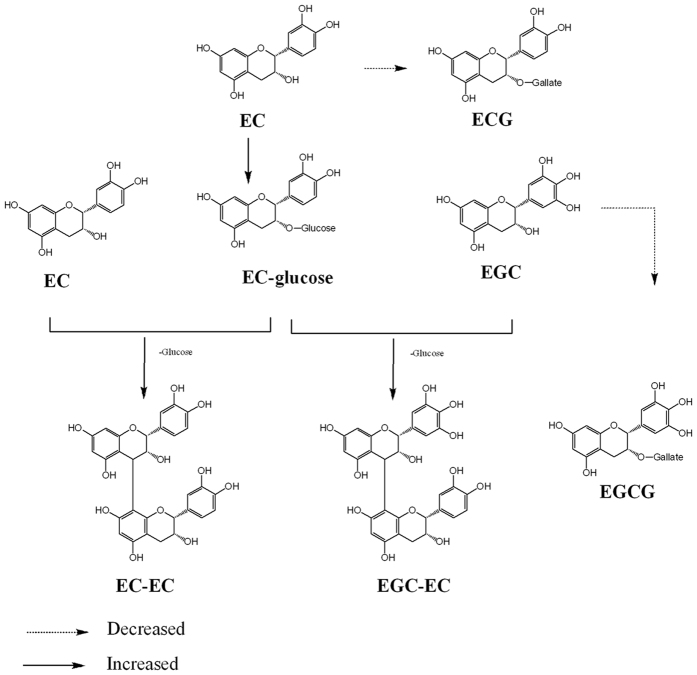
The proposed biosynthesis of procyanidins in the *Camellia* species.

Finally, our quantitative analysis showed that the contents of four procyanidins in *C. tachangensis* were higher than those in *C. sinensis*. We tried to explain the proposed mechanism of increased biosynthesis of procyanidins using chemical information from the LC-MS data. In brief, we proposed the biosynthesis pathway and procyanidins of *Camellia* species, as shown in Fig. 8.

As shown in Table 1, the contents of EC and EGC in *C. tachangensis* were significantly higher compared with that in *C. sinensis*. Correspondingly, the higher level of EC and EGC led to increased contents of procyanidins EC-EC, EGC-EC, ECG-EC and EC-EC-EC in *C. tachangensis*. These results suggested that EC and EGC might be the critical substrates for the biosynthesis of procyanidins in *Camellia* plants. The physicochemical properties of EC and EGC are similar to other flavonoid aglycones. It has poor solubility in water; therefore, it might be transported in the form of their glucoside^31^. The flavan-3-ols are mainly bio-synthesized in the young shoots and leaves of tea plants, while the procyanidins are mostly distributed in the roots^6^. Therefore, it was suggested that (-)-epicatechin glucoside might be an important carrier for transporting (-)-epicatechin from the young shoots of tea.

Consequently, we hypothesised that EC-glucose might be an important substrate for the biosynthesis of procyanidins in *Camellia* species. To test this hypothesis, we performed a correlation analysis of EC-glucose and procyanidins dimers. The correlation of EC-glucose and procyanidins (EC-EC) exactly matched the biosynthesis hypothesis of procyanidins which we supposed. Although the main secondary metabolites of *C. tachangensis* were different from those of *C. sinensis*, EC and EGC were detected in both species. The accurate determination of flavan-3-ols (catechins) also confirmed that the most remarkable difference between *C. tachangensis* and *C. sinensis* was their contents. To study the secondary metabolism of *C. tachangensis*, the upstream and downstream metabolites of EC were determined quantitatively. The results showed that the biosynthesis of galloylated catechins was decreased in *C. tachangensis*, because the proposed competitive biosynthesis of procyanidins were enhanced in *C. tachangensis*.

Furthermore, the contents of galloylated glucose and galloylated quninc acid of *C. tachangensis* were significantly higher than those of *C. sinensis* (p < 0.001) in Supplementary Figure 6, but galloylated catechins were not increased. It was supposed that the glycosylation of EC and EGC competed with the galloylation of EC and EGC, and then the procyanidins were increased but galloylated catechins were decreased.

## Methods

### Plant Materials

Five *Camellia* species were used in this experiment: *C. sinensis*, C. *gymnogyna*, C. *taliensis, C. tachangensis*, and *C. assamica*. The mature leaves of *Camellia* species were collected in Theacease plant garden in Jinghua city, Zhejiang province, China. All of these fresh tea samples were transported in the liquid nitrogen and dried by freezing dry at the temperature of −40°C. Before analysis, these samples were stored at −80 °C.

### Chemicals and Reagents

Gallic acid (GA), caffeine (CAF), EC, (-)-gallocatechin (GC), EGC, (-)-gallocatechin gallate (GCG), EGCG, and ECG standards were purchased from Shanghai Tongtian Biotechnology company and with the purity more than 98%. Procyanidins B_2_ standard was purchased from Shanghai Yuanye Biotechnology company, and with the purity more than 98%. HPLC-grade acetonitrile (CH_3_CN) and distilled water was used as the mobile phase of HPLC. Other reagents were of analytical grade.

### Preparation of Samples for LC-MS Analysis

Dried tea leaves were ground with a mortar. 200 mg of tea leaves were extracted with 10 mL of a mixed solvent comprising MeOH and H_2_O at a ratio of 4:1 (*v/v*). The mixture was extracted for 30 min and then centrifuged at 2600 g for 3 min. Subsequently, 100 μL of the supernatant was transferred to a 1.5 mL Eppendorf tube. After adding 900 μL of methanol, the extracts were filtered through a 0.22-μm poly-tetrafluoroethylene filter for LC-MS analysis.

### The determination of EC-glucose, EGC-glucose and procyanidins by LC-QQQ-MS

To determine the contents of EC-glucose, EGC-glucose and procyanidins dimers and trimer, the fresh mature tea leaves were collected and dried by freezing-dry at −40°C and reduced pressure for 24 hours. The dried tea leaves were milled in the liquid nitrogen. To extract the secondary metabolites, 200 mg of each tea sample was extracted with 10 mL of a mixed solvent comprising MeOH and H_2_O at a ratio of 4:1 (*v/v*). The mixture was extracted for 30 min by ultrasonic treatment at room temperature and then centrifuged at 2600 g for 3 min. The extract was filtered through a 0.22-μm poly-tetrafluoroethylene filter for LC-QQQ-MS analysis, and 3 μL of extract was injected to LC-QQQ-MS for analysis.

Procyanidins B_2_ was used as the reference standard in establishing the calibration curve. The different concentrations of procyanidins B_2_ solution were prepared by continuous diluting the procyanidins B_2_ stock solution with methanol. 3 μL of procyanidins B_2_ standard solutions with different concentration (0.0001, 0.001, 0.01, 0.02 mg/mL) were injected to the LC-QQQ-MS. The chromatographic and mass parameters were listed as below.

LC-QQQ-MS analysis was performed on a UHPLC-ESI-MS system consisting of a Agilent 6460 triple-quadrupolemass spectrometer (Agilent 6460, San Jose, CA, USA) coupled to a Agilent 1260 series HPLC system (Agilent Technologies, Palo Alto, CA, USA) equipped with auto-injector, a quaternary solvent delivery system. Chromatographic separation of black tea extract was conducted using a Acquity UPLC shield RP-18 column (50 mm × 2.1 mm, 1.7 μm) equipped with an Acquity UPLC C_18_ guard column (Waters, Milford, MA, USA) at a flow rate of 0.3 mL/min, and the column was thermostated at 30 °C. The mobile phase consisted of 0.1% formic acid in water (v/v) (A) and acetonitrile (B), and with the gradient elution at 0–5 min: 5–15% B, 5–8 min: 15–30% B, 8–13 min: 30% B, 13–23 min: 30–88% B, 23–28 min: 88–93% B, 28–30 min: 93–93% B, 30–33 min: 93–5% B, 33–35 min: 5% B. The injection volume was 3.0 μL. The entire eluant was sprayed into the mass spectrometer at −3500 kV with nebulizer, sheath and sweep gases set at 70, 20 and 5 arbitrary units, respectively, and desolvation of the solvent droplets was further aided by setting the heated capillary temperature at 350 °C.

### RNA Extraction and qPCR analysis

Total RNA was extracted separately from leaves using the modified cetyl trimethylammonium bromide (CTAB) method^32^. RNA integrity was measured using gel electrophoresis and spectrophotometry (Nanodrop), and the single-stranded cDNAs used for qPCR analysis were synthesized from the RNAs using a Prime-Script™ 1st Strand cDNA Synthesis Kit (Takara, Dalian, China). The primers for *LAR1, LAR2, ANR1, ANR2* were the same as those referenced in a previous publication^6^. Primers were ordered from Sangon Biotech (Shanghai) Co., Ltd. qPCR was performed using the SYBR Green qPCR mastermix (Takara, SYBR Premix Ex TaqII™) at an annealing temperature of 60 °C on a Bio-Rad CFX 96™ real-time PCR system (Bio-Rad), according to the manufacturer’s instructions. The housekeeping gene, glyceraldehyde-3-phosphate dehydrogenase (*GAPDH*) was used as an internal reference gene, and the relative expression was calculated using the 2^−ΔΔCT^ method^33^.

### Molecular cloning of LAR and ANR in C. *tachangensis* and C. *sinensis*

Total RNA was reverse transcribed using the PrimeScript™ Reagent Kit with the gDNA Eraser (TaKaRa). The obtained cDNA was used as the template for subsequent PCR reactions. PCR reaction was performed with F and R primer sets (Supplementary Table 1) with cDNA as the template to amplify these four fragments. The PCR mixture (25 mL) consisted of 100 ng cDNA, 0.5 μl KOD, 2.5 μl dNTP Mixture, 1.5 μl MgSO4, F and R primers and ddH_2_O. A total of 4 μl of PCR products were visualized by electrophoresis on a 1% (w/v) agarose gel. The target DNA band in agarose was cut under UV and fragment was extracted using Gel Extraction Kit. Then these genes were cloned into vector and sequenced. Genes sequencing were performed by Shanghai RuiDi Biological Technology Co. Ltd.

### Statistical analysis

Results were expressed as mean ± SD, with the number of determinations (n = 3) representing separate experiments. Data were evaluated at a 0.05 level of significance with one-way ANOVA with post-hoc testing by Fisher’s protected least significant differences procedure. The correlation coefficient was calculated by SPSS 20.0 (SPSS Inc., Chicago, IL, USA).

## Additional Information

**How to cite this article:** Zhang, L. *et al*. The proposed biosynthesis of procyanidins by the comparative chemical analysis of five *Camellia* species using LC-MS. *Sci. Rep.* **7**, 46131; doi: 10.1038/srep46131 (2017).

**Publisher's note:** Springer Nature remains neutral with regard to jurisdictional claims in published maps and institutional affiliations.

## Supplementary Material

Supplementary Figures and Tables

## Figures and Tables

**Table 1:** The contents of major compounds of *C. sinensis, C. assamica, C. taliensis, C. gymnogyna* and *C. tachangensis* (mg/g) (Mean ± S.d.).

Compound^*a*^	GA	GC	EGC	EC	CAF	EGCG	GCG	ECG
*C. assamica*	0.47 ± 0.01	0.37 ± 0.01	3.74 ± 0.08	1.99 ± 0.04	7.84 ± 0.24	34.73 ± 0.83	0.37 ± 0.01	4.39 ± 0.11
*C. sinensis*	0.44 ± 0.01	3.19 ± 0.16	8.79 ± 0.07	4.16 ± 0.02	6.41 ± 0.06	23.91 ± 0.19	0.19 ± 0.01	4.38 ± 0.04
*C. taliensis*	0.58 ± 0.01	1.43 ± 0.03	ND	ND	0.65 ± 0.16	15.37 ± 0.23	0.14 ± 0.00	3.39 ± 0.04
*C. gymnogyna*	0.10 ± 0.01	ND	1.20 ± 0.09	0.58 ± 0.02	2.34 ± 0.25	0.64 ± 0.06	0.17 ± 0.01	3.99 ± 0.13
*C. tachangensis*	0.82 ± 0.01***	ND	12.01 ± 0.14***	9.50 ± 0.10***	0.13 ± 0.03***	1.48 ± 0.03***	0.32 ± 0.01	0.09 ± 0.01***

ND, not detected, ***p < 0.001 compared with *C. sinensis*. GA, gallic acid; GC, (-)-gallocatechin; (-)-EGC, (-)-epigallocatechin; EC, (-)-epicatechin; CAF, caffeine; EGCG, (-)-epigallocatechin gallate; GCG, (-)-gallocatechin gallate; ECG, (-)-epicatechin gallate.
