# Supplementary material for: The proposed biosynthesis of procyanidins by the comparative chemical analysis of five Camellia species using LC-MS
Source: Sci Rep. 2017 Apr 6;7:46131. doi: 10.1038/srep46131 (PMC5382677; doi:10.1038/srep46131)
Supplement: Supplementary Figures and Tables [file srep46131-s1.pdf]

The proposed biosynthesis of procyanidins by the comparative chemical analysis of five *Camellia* species using LC-MS

Liang Zhang[a†], Yuling Tai[a,b†], Yijun Wang[a], Qilu Meng[a], Yunqiu Yang[a], Shihua Zhang[a], Hua Yang[a], Zhengzhu Zhang[a], Daxiang Li[a], & Xiaochun Wan[a*]

[a] State Key Laboratory of Tea Plant Biology and Utilization, Anhui Agricultural University, Hefei 230036, China

[b] School of Life Science, Anhui Agricultural University, Hefei 230036, China

[†]These authors contributed equally to this work. [*]Correspondence and requests for materials should be addressed to X.C. Wan (email: xcwan@ahau.edu.cn), Tel/Fax: 86-551-85786765

Supplementary Figure 1

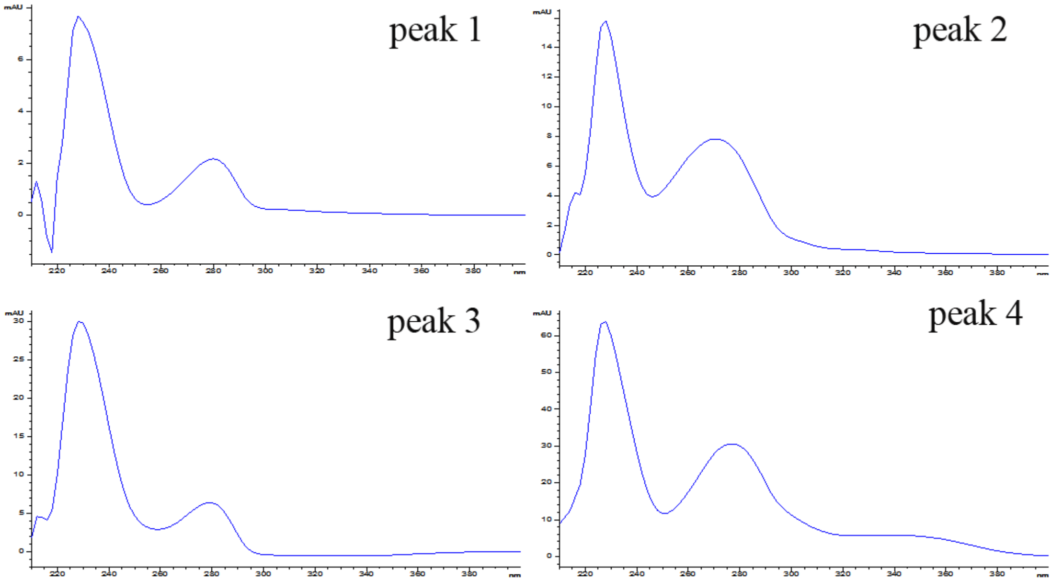

Figure 1 The ultraviolet absorption spectrum for compound 1-4 of *C. tachangensis*

Supplementary Figure 2

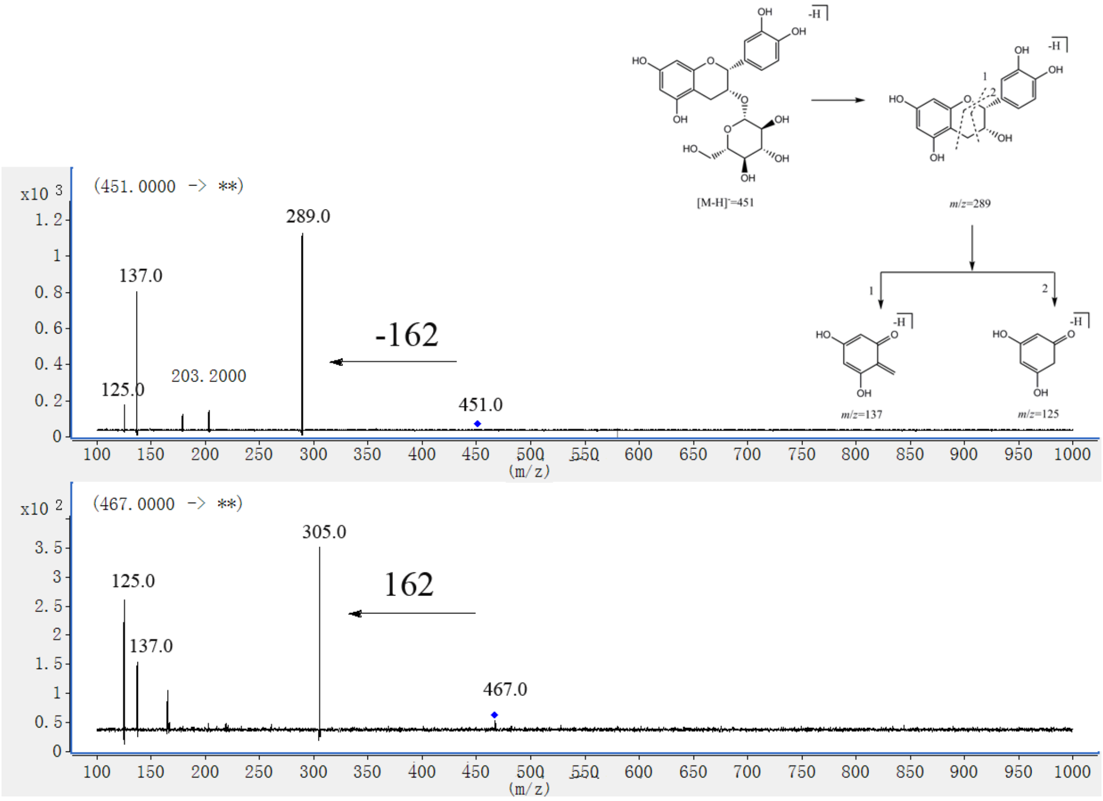

Figure 2 The MS/MS spectrum of m/z at 451 (EC-glucose) and 467 (EGC-glucose)

Supplementary Figure 3

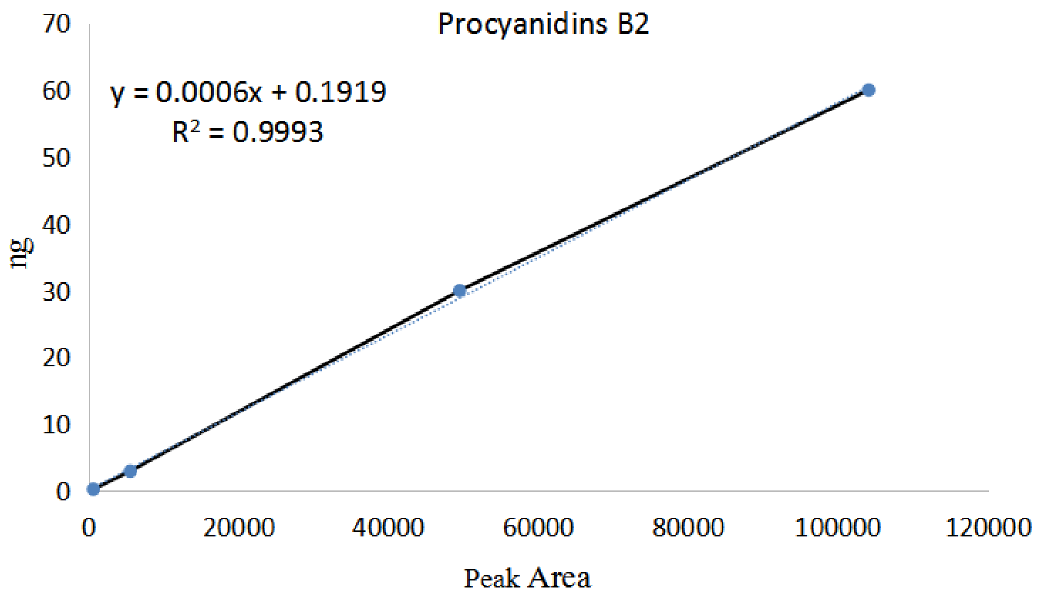

Figure 3 The calibration curve of procyanidins B_2 by LC-QQQ-MS analysis

Supplementary Figure 4

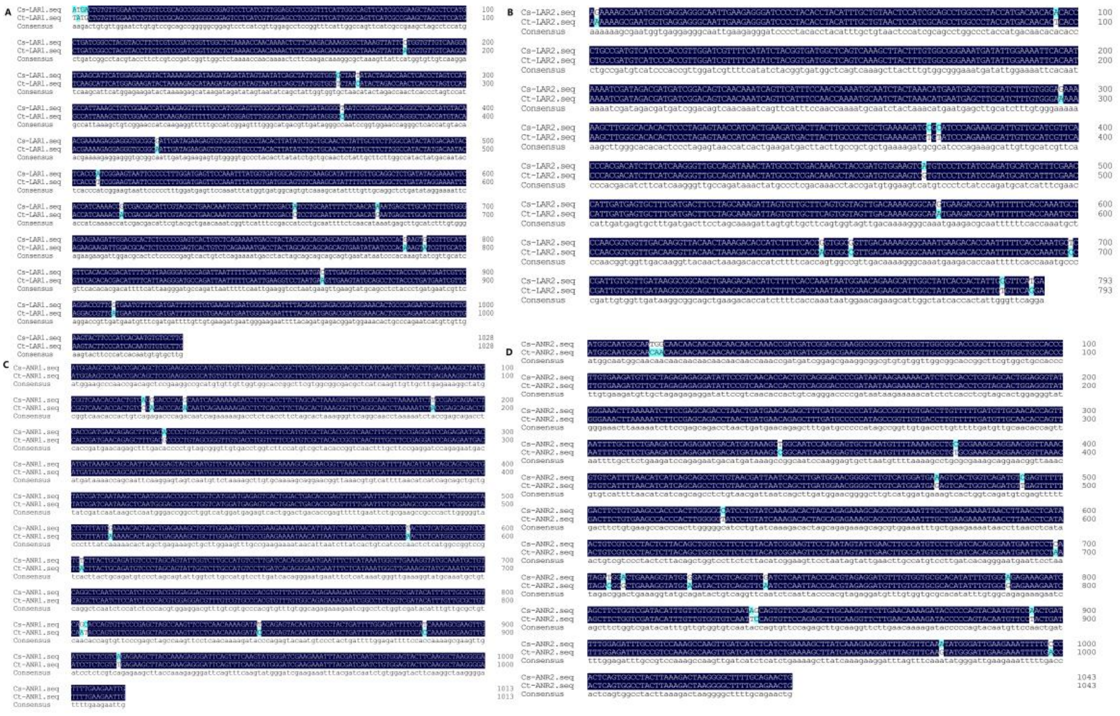

Figure 4 The homology comparison of LAR1 (A), LAR2 (B), ANR1 (C) and ANR2 (D) genes between C. *sinensis* and C. *tachangensis*.

Supplementary Figure 5

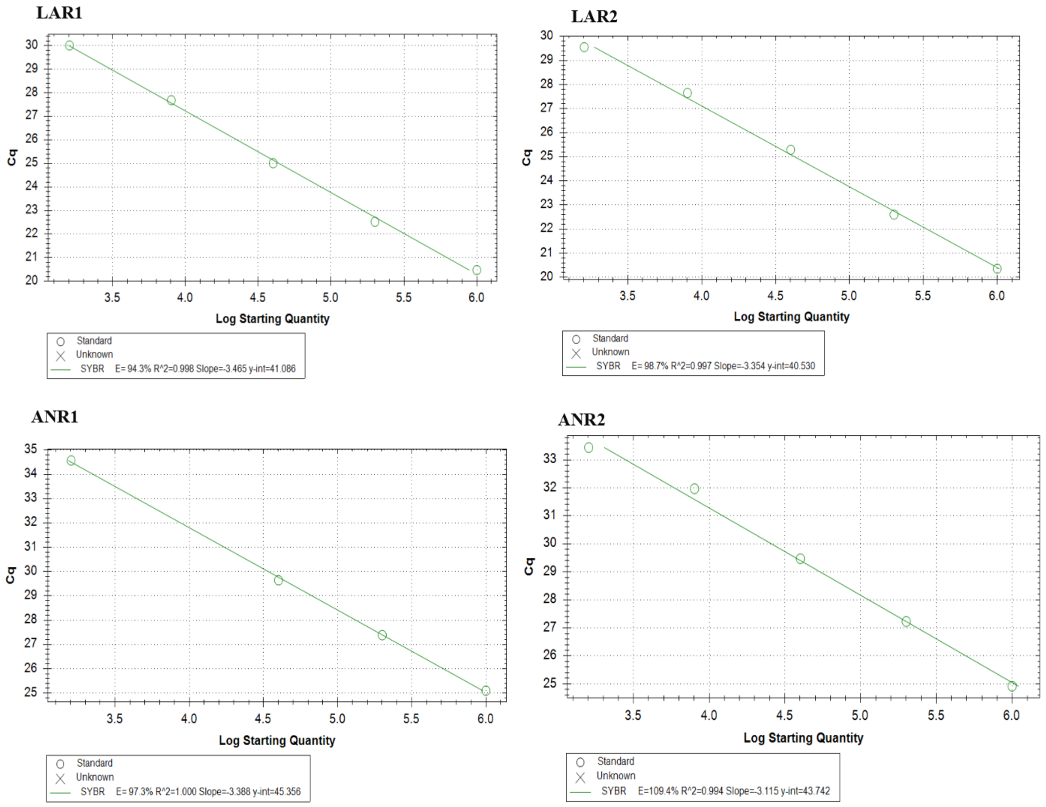

Figure 5 Amplification efficiency of primers for qPCR

Supplementary Table 1

Table 2 Primers for gene cloning

Genes	Primers
ANR1-F	ATGGAAGCCCAACCGACAGCTCCGA
ANR1-R	TCAATTCTTCAAAATCCCCTTAGCC
ANR2-F	ATGGCAATGGCAATGGCAACAACAA
ANR2-R	TCAGTTCTGCAAAAGCCCCTTAGTC
LAR1-F	ATGACTGTGTTGGAATCTGTGTCCG
LAR1-R	CAAGCACACATTGTGATGGGAAGT
LAR2-F	ATGTACAAAGAAAAGCGAATGGTGA
LAR2-R	TCATGAACAAGTAGTGGTGATAGCC

The determination of galloylated glucose, galloylated quinic acid, flavonoid glycosides by LC-QQQ-MS

To determine the contents of galloylated glucose, galloylated quinic acid, flavonoid glycosides, the fresh mature tea leaves were collected and dried by freezing-dry at -40℃ and reduced pressure for 24 hours. The dried tea leaves were milled in the liquid nitrogen. To extract the secondary metabolites, 200mg of each tea sample was extracted with 10 mL of a mixed solvent comprising MeOH and H_2O at a ratio of 4:1 (*v*/*v*). The mixture was extracted for 30 min by ultrasonic treatment at room temperature and then centrifuged at 12000 rpm for 3 min. The extract was filtered through a 0.22-μm poly-tetrafluoroethylene filter for UHPLC-QQQ-MS analysis, and 3 μL of extract was injected to UHPLC-QQQ-MS for analysis.

Rutin was used as the reference standard in establishing the calibration curve. The different concentrations of procyanidins B_2 solution were prepared by continuous diluting the procyanidins B_2 stock solution with methanol. 3 μL of rutin standard solutions with different concentration (0.00013, 0.0013, 0.013, 0.13 mg/mL) were injected to the UHPLC-QQQ-MS (supplementary **Figure 5**). The chromatographic and mass parameters were listed as below.

LC-QQQ-MS analysis was performed on a UHPLC-ESI-MS system consisting of a Agilent 6460 triple-quadrupolemass spectrometer (Agilent 6460, San Jose, CA, USA) coupled to a Agilent 1260 series HPLC system (Agilent Technologies, Palo Alto, CA, USA) equipped with auto-injector, a quaternary solvent delivery system. Chromatographic separation of black tea extract was conducted using a Acquity

UPLC shield RP-18 column (50 mm×2.1 mm, 1.7μm) equipped with an Acquity UPLC C18 guard column (Waters, Milford, MA, USA) at a flow rate of 0.3 mL/min, and the column was thermostated at 30 ℃. The mobile phase consisted of 0.1% formic acid in water (v/v) (A) and acetonitrile (B), and with the gradient elution at 0-5 min: 5-15% B, 5-8min: 15-30% B, 8-13min: 30%B, 13-23 min: 30-88%B, 23-28 min: 88-93%B, 28-30 min: 93-93%B, 30-33min: 93-5%B, 33-35min:5%B. The injection volume was 3.0 μL. The entire eluant was sprayed into the mass spectrometer at -3500 kV with nebulizer, sheath and sweep gases set at 70, 20 and 5 arbitrary units, respectively, and desolvation of the solvent droplets was further aided by setting the heated capillary temperature at 350 ℃.

Through the UHPLC-QQQ-MS, the product ions of galloylated glucose, galloylated quinic acid, kaempferol-glucose, quercetin-glucose, kaempferol-glucose-rhamnose, quercetin-glucose-rhamnose, kaempferol-gulcose-rhamnose-rhamnose, quercetin-glucose-rhamnose-glucose were obtained. All of the flavonoids glycosides compounds showed the product ion *m/z* at either 285 or 301, which are the $[M-H]^-$ ions of kaempferol and quercetin. On the other side, the galloylated-glucose and galloylated quinic acid showed the main product ions *m/z* at 169 and 191, which are the $[M-H]^-$ ions of gallic acid and quinic acid, respectively. The collision energies (CE) were selected at 20 V for galloylated glucose, galloylated quinic acid, kaempferol-glucose, quercetin-glucose, kaempferol-glucose-rhamnose, quercetin-glucose-rhamnose, and 30 V for quercetin-glucose-rhamnose-rhamnose and quercetin-glucose-rhamnose-glucose. Multiple reaction monitoring (MRM) mode was

employed to detect the target compounds by selected product ions from the parent ions (galloylated glucose, 331 → 169; galloylated quinic acid, 343 → 191; kaempferol-glucose, 447 → 285; quercetin-glucose, 463 → 301; kaempferol-glucose-rhamnose, 593→285; quercetin-glucose-rhamnose, 609→301; quercetin-glucose-rhamnose-rhamnose, 755 → 301; and quercetin-glucose-rhamnose-glucose, 771→301.

Supplementary Figure 6

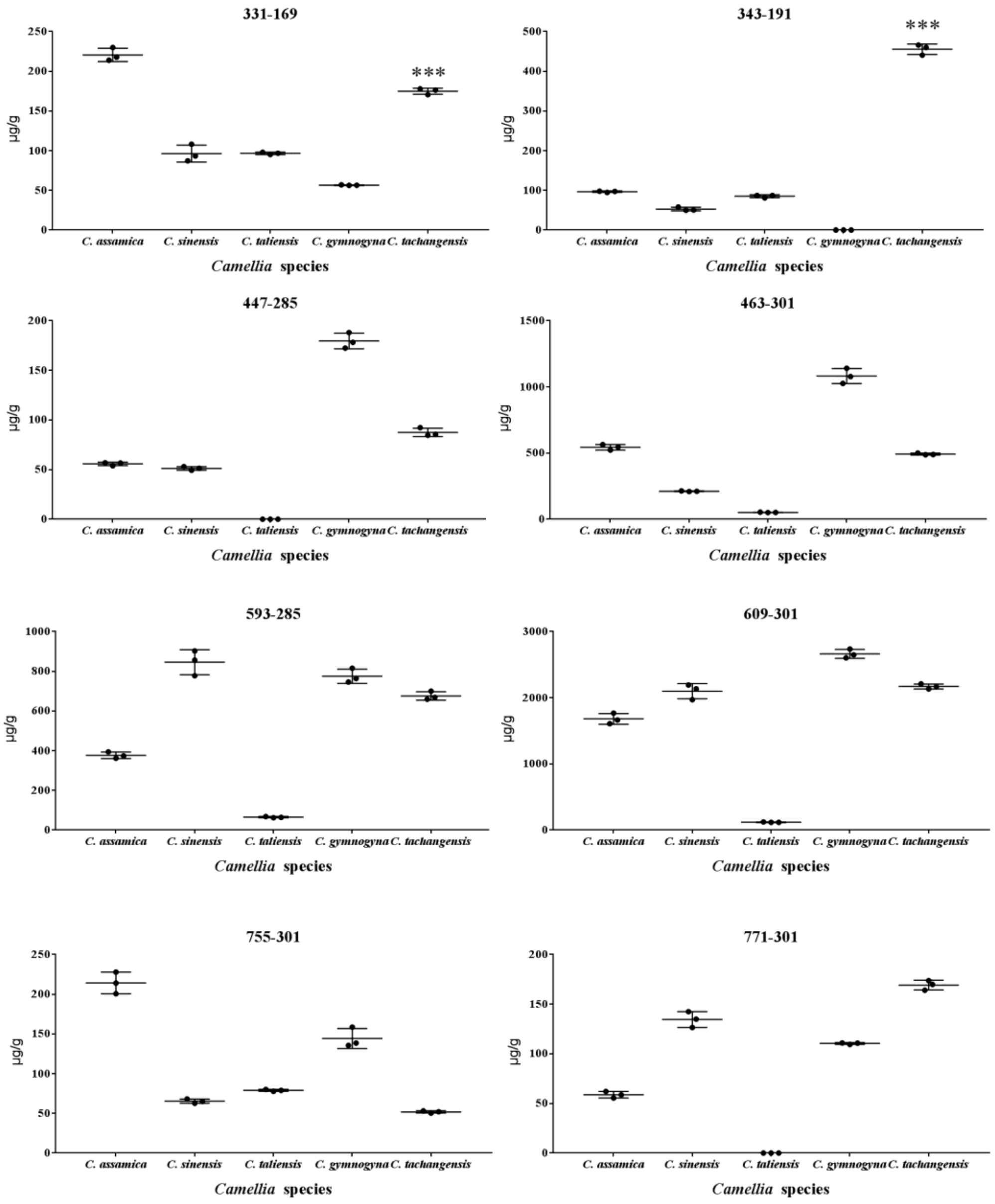

Figure 6 The contents of galloylated glucose, galloylated quinic acid, kaempferol-glucose, quercetin-glucose, kaempferol-glucose-rhamnose, quercetin-glucose-rhamnose, kaempferol-gulcose-rhamnose-rhamnose, quercetin-glucose-rhamnose-glucose in five *Camellia* species by LC-QQQ-MS

***p<0.001 compared with *C. sinensis*

Supplementary Figure 7

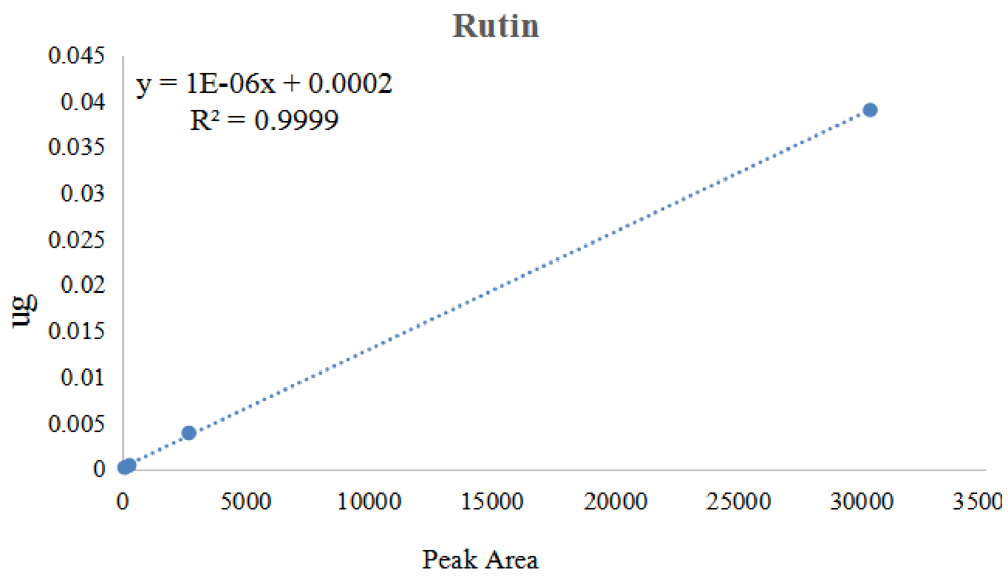

Figure 7 The calibration curve of rutin by LC-QQQ-MS analysis

Supplementary Table 2

Table 2 The Calibration curves of chemical standards of tea

Compounds	Calibration curves	Range (ng)	r
GA	y = 0.000312 x+1.589694	9.08-108.90	0.995
GC	y = 0.004688 x+5.782366	18.42-221.00	0.993
EGC	y = 0.019576 x+68.833375	276.70-3320.00	0.994
EC	y = 0.001152 x + 20.929627	127.22-1526.67	0.995
CAF	y = 0.000267 x + 56.511957	347.00-3470.00	0.999
EGCG	y = 0.000739 x + 217.940254	543.33-5433.30	0.996
GCG	y = 0.000668 x - 2.901100	1.00-200.00	0.991
ECG	y = 0.000477 x + 208.438453	508.00-5080.00	0.997
